# Multi‐decade national cohort identifies adverse pregnancy and birth outcomes associated with acute respiratory illness hospitalisations during the influenza season

**DOI:** 10.1111/irv.13063

**Published:** 2022-10-28

**Authors:** Jazmin Duque, Anna S. Howe, Eduardo Azziz‐Baumgartner, Helen Petousis‐Harris

**Affiliations:** ^1^ Department of General Practice and Primary Health Care, Faculty of Medical and Health Sciences The University of Auckland Auckland New Zealand; ^2^ Abt Associates Inc Atlanta Georgia USA; ^3^ Department of Paediatrics: Child and Youth Health, Faculty of Medical and Health Sciences The University of Auckland Auckland New Zealand; ^4^ School of Health Sciences University of Canterbury Christchurch New Zealand; ^5^ National Center for Immunization and Respiratory Diseases U.S. Centers for Disease Control and Prevention Atlanta Georgia USA

**Keywords:** influenza, maternal, outcomes, pregnant, preterm birth, viruses

## Abstract

**Background:**

Despite the World Health Organization (WHO) recommendation that pregnant women be prioritised for seasonal influenza vaccination, coverage in the Western Pacific Region remains low. Our goal was to provide additional data for the Western Pacific Region about the value of maternal influenza vaccination to pregnant women and their families.

**Methods:**

We conducted a 16‐year retrospective cohort to evaluate risks associated with influenza‐associated maternal acute respiratory infection (ARI) in New Zealand. ARI hospitalisations during the May to September influenza season were identified using select ICD‐10‐AM primary and secondary discharge codes from chapter J00–J99 (diseases of the respiratory system). Cox proportional hazards models were used to calculate crude and adjusted hazard ratios (aHRs) and 95% confidence intervals (CIs).

**Results:**

We identified 822,391 pregnancies among New Zealand residents between 2003 and 2018; 5095 (0.6%) had ≥1 associated ARI hospitalisation during the influenza season; these pregnancies were at greater risk of preterm birth (aHR 1.50, 95% CI 1.39–1.61) and low birthweight (aHR 1.64, 95% CI 1.51–1.79) than pregnancies without such hospitalisations. We did not find an association between maternal ARI hospitalisation and fetal death (aHR 0.96, 95% CI 0.69–1.34) during the influenza season. Maternal influenza vaccination was associated with reduced risk of preterm birth (aHR 0.79, 95% CI 0.77–0.82), low birthweight (aHR 0.87, 95% CI 0.83–0.90) and fetal death (aHR 0.50%, 95% CI 0.44–0.57).

**Conclusion:**

In this population‐based cohort, being hospitalised for an ARI during the influenza season while pregnant was a risk factor for delivering a preterm or a low birthweight infant and vaccination reduced this risk.

## 1 BACKGROUND

Pregnant women with influenza are at increased risk of hospitalisation and intensive care unit (ICU) admission than their non‐pregnant counterparts.^1^, ^2^, ^3^, ^4^ In addition, pregnant women with influenza may be at increased risk of pregnancy‐related complications and poor birth outcomes compared with pregnant women without influenza.^5^, ^6^ Two studies from the 2009 pandemic suggested that hospitalised maternal cases were at increased risk of preterm birth.^7^, ^8^ Whereas these studies observed a significant risk of influenza‐associated hospitalisation among pregnant women, several others did not.^9^, ^10^ In 2017, Fell *et al* ^11^ systematically reviewed studies that reported on maternal influenza and birth outcomes for the World Health Organization (WHO). They concluded that additional high‐quality studies about the risk of preterm birth, small for gestational age and fetal death following maternal influenza virus infection were needed.^12^

Insufficient information about the value proposition for influenza vaccination of pregnant women hampers many vaccination programmes. For example, despite the WHO recommendation that pregnant women be prioritised for seasonal influenza vaccination, coverage in the Western Pacific Region remains suboptimal.^13^ Some of the most significant barriers to immunisation during pregnancy are a lack of information about the benefits and safety of vaccination, insufficient buy‐in from pregnant women's healthcare providers and barriers to preventive care.^13^, ^14^ To substantiate the value of influenza vaccination in preventing more than respiratory illnesses among pregnant women and their infants, we sought to (1) quantify the association between acute respiratory infection (ARI) hospitalisation during the influenza season and adverse birth outcomes and (2) measure the effects of influenza vaccination on pregnancy outcomes. This study was designed as a retrospective cohort study to minimise the potential for bias and to recruit a large enough sample size to obtain statistical power for analysis. Findings from this study can be used by maternity services and antenatal healthcare providers in risk communication about maternal influenza vaccination.

## 2 METHODS

### 2.1 Data sources

The study used New Zealand Ministry of Health National Collections datasets, which are used to support policy, funding and point‐of‐care decision‐making. Data from the National Health Index, National Immunisation Registry, National Maternity Collection, National Minimum Dataset, Mortality Collection and Pharmaceutical Collection were used in the study (Table 1). Immunisation data from Proclaims, a dataset that holds fee‐for‐service payments made to general practitioners for patient visits in New Zealand, were also used.^13^ Every individual in the National Collections datasets has an individual‐level unique identifier (National Health Index number). The National Maternity Collection started in calendar year 2000 and holds data about selected publicly funded maternity and newborn services during pregnancy and up to 3 months after birth sourced from payment claims for lead maternity care visits. The National Minimum Collection started in 1999 and holds inpatient and outpatient public and private hospital discharge data, including clinical information. Mothers were linked to their child(ren) using unique identifiers. The National Mortality Collection provides data for all registered deaths in New Zealand, including fetal deaths.

**TABLE 1:** New Zealand Ministry of Health National Collections datasets used in the study

Data source	Year started	Description	Relevant fields
National Health Index (NHI)	The National Master Patient Index was implemented in 1977 and replaced by the NHI in 1993	Every person who uses health and disability services in New Zealand gets an NHI number assigned for the purposes of identifying every healthcare user.	Encrypted NHI number; date of birth; date of death; New Zealand Deprivation Index 2013 (NZDep‐13) decile; New Zealand resident status; prioritised ethnicity
National Maternity Collection	2000	Selected publicly funded maternity and newborn services during pregnancy and up to 3 months after birth.	Encrypted NHI number; birthweight; gestational age; other data collected during maternity services and inpatient visits across the pregnancy and birth periods pertaining to both fetus/infant and mother
National Minimum Dataset	1999	Inpatient and outpatient public and private hospital discharge data, including clinical information.	Encrypted NHI number; admission and discharge data; ICD‐10 discharge codes
Mortality Collection	1970	Classification of underlying cause of death for all deaths registered in New Zealand and all registered fetal death (stillbirths).	Encrypted NHI number; date of death
National Immunisation Register	2005	All immunisation enrolments and events per the National Immunisation Schedule.	Encrypted NHI number; vaccination date; vaccine type
Pharmaceutical Collection	2012	Claims and payment information that supports the management of pharmaceutical subsidies.	Encrypted NHI number; dispensing date (vaccination date); claim type (vaccine type)

### 2.2 Study population and analytic sample

The study population included all women residents of New Zealand of reproductive age (15–49 years)^15^ who were pregnant anytime between 1 January 2003 and 31 December 2018. The analytic sample only included women whose last menstrual period, date of hospitalisation (if applicable) and date of delivery took place during the 1 January 2003 through 31 December 2018 observation period. Thus, women who were pregnant (by date of last menstrual period) before January 2003 and women who gave birth after December 2018 were excluded from the analytic dataset.

To address the study objectives, we examined each individual pregnancy and its birth outcomes. Pregnancy was the unit of observation. We defined ARI as a maternal hospitalisation that took place during the influenza season with select respiratory illness ICD‐10‐AM (The International Statistical Classification of Diseases and Related Health Problems, Tenth Revision, Australian Modification) primary or secondary discharge codes as described below. A woman could have been pregnant more than once during the observation period and counted as having been hospitalised with influenza‐related ARI during one of her pregnancies and not during the other.

For the mothers in the study, we first identified all hospital admissions at any gestation with primary and secondary ICD‐10‐AM discharge codes from chapter J00–J99 (diseases of the respiratory system). Second, we excluded hospitalisations due to chronic respiratory conditions (ICD‐10‐AM codes J30–39, J60–70, J80–84, J90–94, J95 and J97–99)^16^ that might not have been precipitated by ARI. Finally, the exposed cohort consisted of pregnancies with at least one hospitalisation with primary and secondary ARI ICD‐10‐AM discharge codes that took place during the May to September influenza season when ARIs were most likely to be associated with influenza illness.^17^ For simplicity, we will henceforth refer to a maternal ARI hospitalisation that occurred during the influenza season as a ‘maternal ARI hospitalisation’. The unexposed referent cohort was selected from the same population as the exposed cohort but consisted of pregnancies with no history of ARI hospital admission (per select ICD‐10‐AM codes just described) during the influenza season. The unexposed referent cohort could include women who had been hospitalised during pregnancy for other reasons and women who had an ARI without hospitalisation.

### 2.3 Data analysis

SAS® statistical software Version 9.3 (SAS Institute Inc., Cary, NC, USA) were used for all aspects of data analysis. Univariate analyses were used to describe the study population by demographic and other baseline characteristics (i.e., age group at pregnancy, ethnicity, socioeconomic deprivation, region, smoking status, parity, sex of the baby for singleton pregnancies and body mass index [BMI] before pregnancy). Individuals selected only one priority ethnicity (i.e., Māori, Pacific, Asian, Other and NZ European) even if they identified with several so that the ethnicity used in analysis was prioritised. The New Zealand Deprivation Index 2013 (NZDep‐13) is a measure of socioeconomic status at the smallest geographic area called a ‘meshblock’ defined by Statistics New Zealand.^18^ It combines census data related to income, housing, family structure and employment, among others. The NZDep‐13 groups deprivation scores into deciles, with 10 being the highest level of deprivation. Smoking status was determined by combining variables for report of tobacco use and participation in tobacco use counselling. If smoking status was missing, the woman was considered to be a non‐smoker for that pregnancy.

Bivariate analyses were used to estimate risk ratios (RRs) and 95% confidence intervals (CIs) to describe associations between baseline characteristics and maternal ARI hospitalisation (exposure). Baseline characteristics were assessed as potential effect modifiers and confounders in the relationship between exposure and outcomes of interest to adjust for in multivariate regression models. Variables that could be controlled for in the study included maternal age, ethnicity, deprivation index, region, prior births, sex of baby, smoking status and obesity status. Maternal age was first stratified into 5‐year age groups and age groups that did not have a statistically significant difference in maternal ARI hospitalisation risk were collapsed to improve study power and find meaningful associations between independent variables and outcomes. In New Zealand, women older than 35 years of age are considered to be at higher risk of developing pregnancy‐related complications.^19^ Resident status was first determined using the National Health Index and, if missing, imputed from the National Minimum Data.

Hazard ratios and 95% CIs for maternal ARI hospitalisation and adverse outcomes were estimated using Cox proportional hazards models. Person‐time contributed to the follow‐up period was defined as the total person‐weeks a woman was pregnant and at risk of getting an influenza virus infection. The outcomes of interest were classified into those related to pregnancy (i.e., fetal death and preterm birth) and birth (low birthweight). Fetal death was first identified using the National Maternity Collection and cross‐checked with deaths data from the National Mortality Collection. The same demographic and other baseline characteristics (i.e., age group at pregnancy, ethnicity, socioeconomic deprivation, region, smoking status, parity, sex of the baby for singleton pregnancies and BMI before pregnancy) used in the main analysis were considered prior to regression modelling. The primary model was adjusted for maternal age, ethnicity and smoking status. The PROC PHREG RANDOM statement was used to adjust for clustering due to between‐person correlation among pregnancies.

### 2.4 Supplemental analysis

We conducted a supplemental analysis to examine the potential protective effect of maternal influenza immunisation on mothers and babies using data from years 2010 through 2018. Analysis was limited to pregnancies in 2010 through 2018, to coincide with the approval of government‐funded influenza vaccinations for pregnant women in New Zealand.^20^ Crude and adjusted hazard ratios (aHRs) were estimated accounting for time‐varying influenza circulation, maternal vaccination status and each outcome of interest (i.e., ARI hospitalisation, fetal death, preterm birth and low birthweight). Time contributed for the exposed cohort (influenza vaccinated) started at whichever of the following events occurred last: start of influenza season, date of vaccination or start of pregnancy. Time contributed for the exposed cohort ended at whichever of the following events occurred first: end of the influenza season, birth or end of pregnancy. The model was adjusted for maternal age, ethnicity, smoking status and clustering due to between‐person correlation among pregnancies. Influenza vaccination status was assessed using three data sources: National Immunisation Registry, Pharmaceutical Collection and General Practice Claims (Proclaims). In New Zealand, the National Immunisation Registry is considered the gold standard to assess the status of scheduled vaccines such as maternal influenza vaccines and is supplemented by the Pharmaceutical Collection and Proclaims, which record private purchase vaccinations.^21^

### 2.5 Ethics approval

The study was approved by the University of Auckland Human Participants Ethics Committee (Protocol Number 021530).

## 3 RESULTS

### 3.1 Study cohort

We identified 822,391 pregnancies among New Zealand residents of reproductive age during 1 January 2003 to 31 December 2018. The average maternal age was 29 years (median 30; interquartile range [IQR] 25–34). More than half of the study population (53%) reported being of European or other non‐Asian prioritised ethnicity, 27% Māori, 11% Asian and 10% Pacific. Twenty‐seven percent of pregnancies occurred in mothers of the highest levels of deprivation (NZDep‐13 Index Levels 9–10). Most of the pregnancies occurred in women living in the Northern, Midland and Central regions, with only 19% in the South Island. Approximately 19% of women reported smoking during pregnancy and 40% were primigravids. There were 483,792 women who were pregnant once, 238,290 women who were pregnant twice, 72,666 women who were pregnant thrice and 27,643 women who were pregnant four or more times during follow‐up (Table S1). A total of 806,130 (98.6%) singleton pregnancies were included in the study (Table S2).

### 3.2 Risk of maternal ARI hospitalisation during influenza season

**FIGURE 1:**
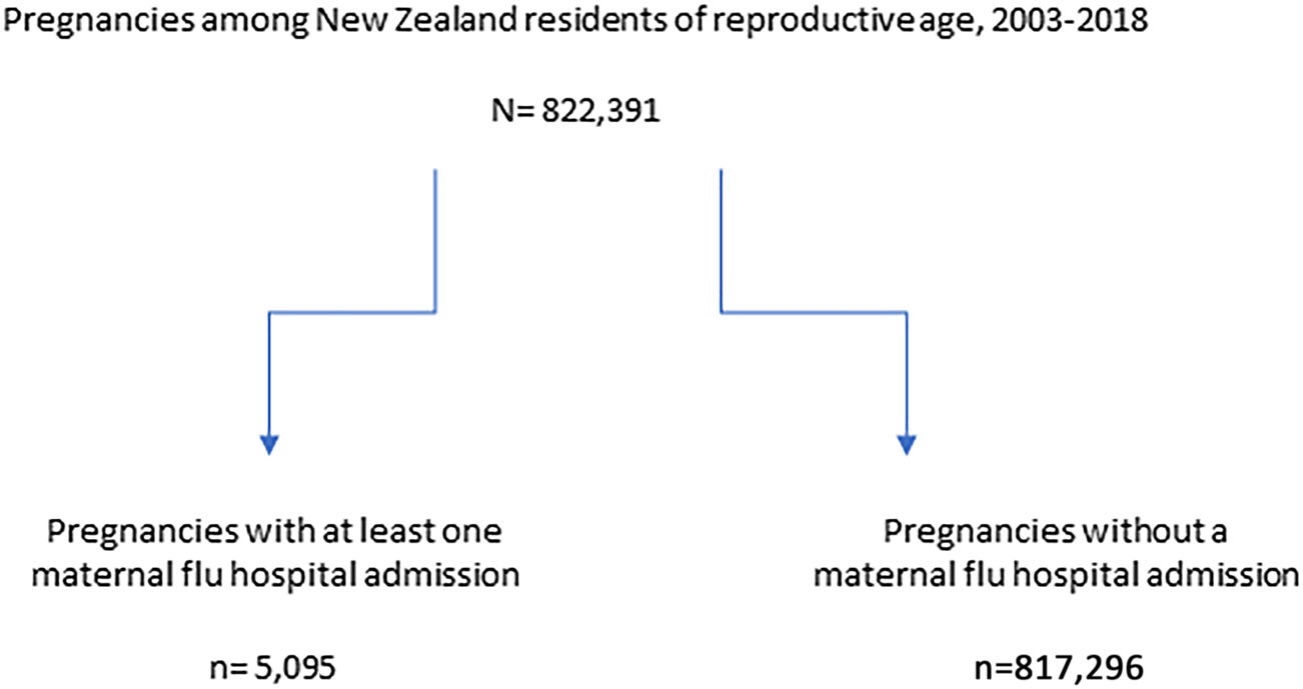
Analytic sample flow diagram. There were N = 882,705 pregnancies for years 2003–2018, but n = 59,478 occurred in women who were not New Zealand residents and 836 in women not of reproductive age (15–49 years).

Only 0.6% (n = 5095) of the pregnancies had at least one maternal ARI hospitalisation during the influenza season and 99.4% (n = 817,296) did not (Figure 1). The risk of maternal ARI varied significantly across the study population by baseline characteristics (Table 2). Older women had a higher risk of maternal ARI hospitalisation. Women aged 45–49 years had 2.35 times the risk (95% CI 1.51–3.63) of being hospitalised than women aged 25–34 years. Similarly, women who primarily identified as Māori and Pacific were also more likely to have maternal ARI hospitalisations. Compared with women whose prioritised ethnicity was European or other, Māori women had 1.83 (95% CI 1.71–1.95) and Pacific women had 2.31 (95% CI 2.13–2.51) times higher risk of ARI hospitalisation during pregnancy compared with women of European or other non‐Asian descent. Women who lived in the most deprived socioeconomic conditions (NZDep‐13 Index Levels 9–10) had twice the risk (95% CI 1.78–2.16) of influenza ARI hospitalisation during pregnancy than women who lived in least deprived conditions (NZDep‐13 Index Levels 1–2). Maternal ARI hospitalisations varied by region (p < 0.01), with the lowest risk being observed in the South Island. Similarly, women who gave birth for the first time (primipara) had a significantly lower risk of influenza hospitalisation than women who were multiparous (RR 0.78, 95% CI 0.74–0.83).

**TABLE 2:** Baseline characteristics of all pregnant women residents of New Zealand, 2013–2018

	Maternal ARI hospitalisation		Risk ratio (95% CI)	Total
	Hospitalised (column %)	Not hospitalised (column %)		(column %)
Total number of pregnancies	n = 5095	n = 817,296		N = 822,391
Age at delivery in years				
15–24	1597 (31.3)	193,307 (23.7)	**1.51 (1.42–1.61)**	194,904 (23.7)
25–34	2451 (48.1)	449,237 (54.9)	1.0	451,688 (54.9)
35–44	1027 (20.2)	173,200 (21.2)	**1.09 (1.01–1.17)**	174,227 (21.2)
45–49	20 (0.3)	1552 (0.2)	**2.35 (1.51–3.63)**	1572 (0.2)
Ethnicity (prioritised)^a^				
Māori	1809 (35.5)	216,490 (26.5)	**1.83 (1.71–1.95)**	218,299 (26.6)
Pacific	836 (16.4)	78,866 (9.6)	**2.31 (2.13–2.51)**	79,702 (9.7)
Asian	486 (9.5)	91,052 (11.1)	**1.17 (1.06–1.29)**	91,538 (11.1)
European/Other	1963 (38.5)	430,504 (52.7)	1.0	432,467 (52.6)
New Zealand deprivation index^b^				
9–10 (most deprived)	1839 (36.3)	216,460 (26.6)	**1.96 (1.78–2.16)**	218,299 (26.7)
7–8	1205 (23.7)	183,813 (22.6)	**1.52 (1.37–1.68)**	185,018 (22.6)
5–6	863 (17.0)	156,579 (19.3)	**1.28 (1.15–1.42)**	157,442 (19.3)
3–4	642 (12.7)	134,245 (16.5)	1.11 (0.99–1.24)	134,887 (16.5)
1–2 (least deprived)	524 (10.3)	121,568 (15.0)	1.0	122,092 (14.9)
Region^c^				
Northern	2366 (47.0)	310,805 (39.0)	**1.58 (1.46–1.72)**	313,171 (39.1)
Midlands	1040 (20.7)	169,924 (21.3)	**1.27 (1.16–1.40)**	170,964 (21.3)
Central	884 (17.6)	161,569 (20.3)	1.14 (1.03–1.26)	162,453 (20.3)
South Island	740 (14.7)	154,269 (19.4)	1.0	155,009 (19.3)
Primipara^d^				
Yes	1647 (33.8)	312,917 (39.6)	**0.78 (0.74–0.83)**	314,564 (39.6)
No	3224 (66.2)	476,979 (60.4)	1.0	480,203 (60.4)
Sex of baby (singleton pregnancies only)^e^				
Male	2453 (50.3)	408,432 (51.3)	0.96 (0.90–1.01)	410,885 (51.3)
Female	2429 (49.7)	386,931 (48.7)	1.0	389,360 (48.7)
Smoking status				
Smoker	1544 (30.3)	150,614 (18.4)	**1.92 (1.81–2.03)**	152,158 (18.5)
Non‐smoker	3551 (69.7)	666,682 (81.6)	1.0	670,233 (81.5)
Body mass index (BMI) before pregnancy^f^				
Obese (BMI ≥ 30)	1440 (37.7)	136,038 (23.4)	**1.97 (1.84–2.10)**	137,478 (23.5)
Not obese (BMI < 30)	2382 (62.3)	445,284 (76.6)	1.0	447,666 (76.5)

Abbreviations: ARI, acute respiratory infection; CI, confidence interval.

^a^ Ethnicity missing: with a maternal influenza hospitalisation n = 1, without a maternal influenza hospitalisation n = 384.

^b^ Deprivation index missing: with a maternal influenza hospitalisation n = 22, without a maternal influenza hospitalisation n = 4631.

^c^ Region missing: with a maternal influenza hospitalisation n = 65, without a maternal influenza hospitalisation n = 21,359.

^d^ Primipara missing: with a maternal influenza hospitalisation n = 224, without a maternal influenza hospitalisation n = 27,400.

^e^ Sex of baby for singleton pregnancies only (n = 806,130). Missing: with a maternal influenza hospitalisation n = 49, without a maternal influenza hospitalisation n = 5836.

^f^ BMI before pregnancy missing: with a maternal influenza hospitalisation n = 1273, without a maternal influenza hospitalisation n = 235,974.

Values that are bolded represent statistically significant point estimates (*p* < 0.05).

Singleton pregnancies carrying a male baby had a similar risk of maternal ARI hospitalisation than singleton pregnancies carrying a female baby (RR 0.96, 95% CI 0.90–1.01). Smokers and women who were obese (BMI ≥ 30) before pregnancy had higher maternal ARI hospitalisations than non‐smokers and women with a BMI < 30 (RR 1.92, 95% CI 1.81–2.03 and RR 1.97, 95% CI 1.84–2.10, respectively).

### 3.3 Maternal ARI hospitalisations and risk of adverse pregnancy and birth outcomes

Maternal ARI hospital admission during influenza season was associated with a greater risk of adverse pregnancy and birth outcomes in multivariate adjusted models compared with pregnancies without maternal ARI hospitalisation during the influenza season (Table 3). After controlling for maternal age, ethnicity, smoking status and clustering, mothers who were hospitalised with maternal ARI were at greater risk of preterm birth (incidence rate 70.3 vs. 47.4 per 10,000 person‐weeks; aHR 1.50, 95% CI 1.39–1.61) and low birthweight (incidence rate 61.5 vs. 33.1 per 10,000 person‐weeks; aHR 1.64, 95% CI 1.51–1.79) than women without maternal ARI hospitalisations during the influenza season. A total of 5633 fetal deaths were detected in the cohort, 34 of which occurred in women who had a maternal ARI hospital admission. The incidence of stillbirth in pregnancies among the hospitalised cohort was similar to that among the unexposed (incidence rate 3.7 vs. 4.1 per 10,000 person‐weeks; aHR 0.96, 95% CI 0.69–1.34).

**TABLE 3:** History of acute respiratory infection hospitalisation while pregnant and risk of adverse pregnancy and birth outcomes in New Zealand, 2003–2018

	Maternal ARI hospitalisation				Hazard ratio (95% CI)	Adjusted hazard ratio^a^ (95% CI)
	Hospitalised (column %)	Incidence rate per 10,000 person‐weeks (95% CI)	Not hospitalised (column %)	Incidence rate per 10,000 person‐weeks (95% CI)		
Pregnancy outcomes						
Fetal death	34 (0.7)	3.7 (2.6–5.1)	5599 (0.7)	4.1 (4.0–4.2)	0.97 (0.69–1.36)	0.96 (0.69–1.34)
Birth outcomes						
Preterm birth (<37 weeks of gestation)	643 (12.6)	70.3 (65.1–75.9)	64,948 (8.0)	47.4 (47.0–47.8)	**1.59 (1.48–1.71)**	**1.50 (1.39–1.61)**
Low birthweight (<2500 g)^b^	562 (11.1)	61.5 (56.6–66.7)	45,457 (5.8)	33.1 (32.9–33.5)	**1.90 (1.76–2.05)**	**1.64 (1.51–1.79)**

Abbreviations: ARI, acute respiratory infection; CI, confidence interval.

^a^ Adjusted for maternal age, ethnicity, smoking status and clustering due to between‐person correlation among pregnancies.

^b^ Birthweight missing: with a maternal influenza hospitalisation n = 186, without a maternal influenza hospitalisation n = 50,275.

Values that are bolded represent statistically significant point estimates (*p* < 0.05).

### 3.4 Possible protective effect of maternal influenza vaccination in the prevention of adverse outcomes

We found an association between influenza vaccination status and adverse pregnancy and birth outcomes (Table 4). After controlling for maternal age, ethnicity and smoking status, mothers who were vaccinated during pregnancy seemed to have lower risk of fetal death (incidence rate 1.0 vs. 1.9 per 10,000 person‐weeks; aHR 0.50%, 95% CI 0.44–0.57), preterm birth (incidence rate 15.5 vs. 20.3; aHR 0.79, 95% CI 0.77–0.82) and low birthweight (incidence rate 1.1 vs. 1.3; aHR 0.82, 95% CI 0.83–0.90). Unadjusted analysis resulted in no statistically significant difference in ARI hospitalisation during the influenza season between mothers who received an influenza vaccination during pregnancy and mothers who did not. In adjusted analysis, however, mothers who were vaccinated against influenza during their pregnancy had a slightly higher risk of ARI hospitalisation than unvaccinated mothers (incidence rate 1.1 vs 1.2; aHR 1.17, 95% CI 1.06–1.29).

**TABLE 4:** Influenza vaccination during pregnancy and risk of adverse pregnancy and birth outcomes in New Zealand, 2010–2018

			Influenza vaccination during pregnancy				Hazard ratio (95% CI)	Adjusted hazard ratio^a^ (95% CI)
			Vaccinated (column %)	Incidence rate per 10,000 person‐weeks (95% CI)	Not vaccinated (column %)	Incidence rate per 10,000 person‐weeks (95% CI)		
Pregnancy outcomes								
ARI hospitalisation during the influenza season			712 (0.8)	1.1 (0.8–1.4)	3215 (0.8)	1.2 (0.9–1.3)	1.04 (0.94–1.15)	1.17 (1.06–1.29)
Fetal death			244 (0.4)	1.0 (0.9–1.1)	2847 (0.8)	1.9 (1.8–2.0)	**0.50 (0.44–0.57)**	**0.50 (0.44–0.57)**
Birth outcomes								
Preterm birth (<37 weeks of gestation)			3895 (6.1)	15.5 (15.0–16.0)	29,825 (8.0)	20.3 (20.1–20.6)	**0.76 (0.74–0.79)**	**0.79 (0.77–0.82)**
Low birthweight (<2500 g)			2692 (4.4)	1.1 (1.0–1.1)	18,869 (5.4)	1.3 (1.3–1.3)	**0.82 (0.79–0.85)**	**0.87 (0.83–0.90)**

Abbreviations: ARI, acute respiratory infection; CI, confidence interval.

^a^ Adjusted for maternal age, ethnicity, smoking status and clustering due to between‐person correlation among pregnancies.

Values that are bolded represent statistically significant point estimates (*p* < 0.05).

## 4 CONCLUSION

Our analysis of 16 years of New Zealand data demonstrates that pregnant women with ARI hospitalisation during the influenza season had a higher risk of adverse birth outcomes; conversely, pregnant women who were influenza vaccinated were at lower risk of experiencing these outcomes. Our study also explored the relationship between influenza vaccination during pregnancy and ARI hospitalisation during the influenza season, and adverse pregnancy and birth outcomes. Maternal influenza vaccination did not decrease the risk of ARI hospitalisation for pregnant women.

The main limitation of this study is that although we were able to quantify the hazard of adverse pregnancy and birth outcomes among women hospitalised with ARI during the influenza season, we were unable to conduct similar analyses for women seeking care for ARI in ambulatory settings. It is possible that hospitalised pregnant women may be more likely to have gestational diabetes and other underlying medical conditions, which place them at risk for preterm delivery regardless of whether they are infected with a respiratory virus or not. We could not include underlying medical conditions other than obesity in the analyses because of data limitations. During epidemic periods, a greater proportion of hospitalised pregnant women could have had an influenza‐associated ARI, but it might be that their diabetes, and not the ARI, for example, placed them at higher risk of an adverse birth outcome. However, it is also possible that influenza virus infection triggered cytokine dysregulation and exacerbated underlying medical conditions that disproportionately affected pregnant women and led to hospitalisation.^22^ Associations between maternal ARI and adverse pregnancy outcomes have been documented in the literature since the 1918 influenza A(H1N1) pandemic.^23^ A 13‐year retrospective cohort study from Nova Scotia, Canada, observed a higher risk of small for gestational age among mothers who had been hospitalised for a respiratory illness during influenza season.^24^ A 2021 study in three middle‐income countries also found an association between maternal influenza infection and a significant reduction in birthweight among full‐term babies.^25^

Another limitation of this study is that maternal ARI hospitalisations were not laboratory confirmed for influenza, and it is likely that our study captured ARI hospitalisations associated with other respiratory pathogens that co‐circulate during the influenza season, not just influenza. Less than half of specimens tested from patients hospitalised with ARI during the influenza season test positive for influenza, and the other half test positive for non‐influenza viruses, like adenovirus, respiratory syncytial virus and rhinovirus.^17^ Although laboratory confirmation would have been preferable, there is increasing evidence that maternal ARI for a variety of aetiologies, including influenza and SARS‐CoV‐2‐associated ARI,^26^ can adversely affect a pregnancy. A recent multi‐country retrospective cohort analysis found that women with acute respiratory or febrile illness hospitalisations, regardless of whether they were influenza associated or not, were more likely to have preterm births and low birthweight babies.^27^

This study provides comprehensive estimates of maternal ARI hospital admission rates during the influenza season among residents of New Zealand. We found significantly increased risk of maternal ARI hospitalisations among the older (45–49 years) age group. Pregnancies among women older than 35 years are regarded as more at risk of pregnancy‐related complications,^19^ and there was more than a twofold risk of maternal ARI hospitalisation in women aged 45–49 years compared with women aged 35–44 years. Our findings also suggest that the risk of hospitalisation was markedly higher in Māori and Pacific, as well as women in the most deprived socioeconomic groups. These findings are consistent with other New Zealand publications from the 2009 influenza A(H1N1) pandemic^28^ and enhanced population‐based influenza surveillance (SHIVERS) in the Auckland Region.^29^ Maternal ARI hospitalisations were less common in the South Island region than in the North Island (Northern, Midlands and Central regions). We speculate that this was because of ethnic differences in each geographic region. Eighty‐five percent of South Islanders identified as European ethnicity compared with 66% of North Islanders.^30^

Our finding that influenza‐vaccinated mothers had almost half the incidence of fetal death compared with unvaccinated mothers is consistent with that observed in Australia.^31^ The proportion of stillbirths that can be attributed to influenza infection remains unknown so it is unclear how many fetal deaths could be prevented with maternal vaccination. Influenza vaccination during pregnancy decreased the risk of preterm birth and low birthweight in our cohort. In a systematic review and meta‐analysis of five studies, Nunes *et al* observed a similar protective effect between seasonal influenza vaccination during pregnancy and adverse birth outcomes.^32^ A pooled analysis of three randomised control trials found no association between maternal vaccination and stillbirth, preterm birth, low birthweight and small for gestation age although it did find that maternal influenza immunisation was protective against low birthweight (RR 0.85, 95% CI 0.74–0.96) in one of the sites.^33^ Our findings can be used by maternity services and antenatal healthcare providers to develop risk communication messages about the value of maternal influenza vaccination in preventing influenza illnesses and their complications among pregnant women and their infants.^34^

Despite the WHO recommendation that pregnant women be prioritised for seasonal influenza vaccination, coverage in the Western Pacific Region remains suboptimal.^13^, ^35^, ^36^ A retrospective cohort study using National Collection datasets found that although influenza vaccination seems to be increasing among pregnant women in New Zealand, only 31% had been vaccinated in 2018.^13^ Studies have documented that influenza vaccines are safe and effective at preventing influenza in pregnant women and infants.^33^ This highlights the importance of conveying the value of maternal influenza vaccination to families in New Zealand and ensuring easy access to vaccines.

This study used data on a national cohort of pregnant women over the span of 16 years available from New Zealand's Ministry of Health National Collection datasets. Our findings from this retrospective cohort analysis indicate that maternal ARI hospitalisations during the influenza season are associated with preterm birth and low birthweight and that influenza vaccination may help prevent these outcomes. Moreover, we found that maternal influenza vaccination was linked to lower rates of fetal death. Findings that support the morbidity, and fetal and infant mortality associated with ARI and influenza infection, along with the benefits of influenza vaccine in this group, are useful to better establish the value proposition of influenza vaccines in preventing respiratory illnesses and related complications. The value of influenza vaccination, in this case, goes beyond preventing mostly self‐limited ARIs and seems to extend to the prevention of expensive and potentially deadly birth outcomes during influenza epidemic periods. Improving understanding, access and provider recommendations relating to maternal influenza immunisation in New Zealand could prove useful in improving pregnancy and birth outcomes.

## AUTHOR CONTRIBUTIONS

**Jazmin Duque:** Conceptualization; formal analysis; investigation; methodology; project administration; resources; software; validation. **Anna S. Howe:** Conceptualization; formal analysis; investigation; methodology; resources; supervision; validation. **Eduardo Azziz‐Baumgartner:** Conceptualization; formal analysis; investigation; methodology. **Helen Petousis‐Harris:** Conceptualization; formal analysis; investigation; methodology; resources; supervision.

## CONFLICTS OF INTEREST

H. Petousis‐Harris reports grant funding from the New Zealand Health Research Council, CDC and the New Zealand Ministry of Health outside this work. No other potential conflicts of interest were disclosed.

### PEER REVIEW

The peer review history for this article is available at https://publons.com/publon/10.1111/irv.13063.

## Supporting information


**Table S1.** Total number of pregnancies per woman during the study period
**Table S2.** Plurality of pregnancies included in the study

## Data Availability

The data that support the findings of this study are available from New Zealand's Ministry of Health. Restrictions apply to the availability of these data, which are governed by data protection and privacy legislation. Data may be requested at https://www.health.govt.nz/nz-health-statistics/access-and-use/data-request-form.
