# Supplementary material for: Multi‐decade national cohort identifies adverse pregnancy and birth outcomes associated with acute respiratory illness hospitalisations during the influenza season
Source: Influenza Other Respir Viruses. 2022 Oct 28;17(1):e13063. doi: 10.1111/irv.13063 (PMC9835450; doi:10.1111/irv.13063)
Supplement: Supplementary file 1 — Table S1. Total number of pregnancies per woman during the study period Table S2. Plurality of pregnancies included in the study [file IRV-17-e13063-s001.docx]

Supplemental Table 1. Total number of pregnancies per woman during the study period

|  | Number | Percent |
| --- | --- | --- |
| Pregnancies per woman |  |  |
| 1 | 483,792 | 58.8 |
| 2 | 238,290 | 29.0 |
| 3 | 72,666 | 8.8 |
| 4 | 19,597 | 2.4 |
| 5 | 5,551 | 0.7 |
| 6 | 1,770 | 0.2 |
| 7 | 557 | 0.1 |
| >7 | 168 | <0.1 |

Supplemental Table 2. Plurality of pregnancies included in the study

|  | Number | Percent |
| --- | --- | --- |
| Plurality |  |  |
| Singleton | 806,130 | 98.6 |
| Twin | 11,609 | 1.4 |
| Multiple | 150 | <0.1 |

Missing n=4,502
